# Endovascular and Endoscopic Treatment for Primary Aortoduodenal Fistula: A Case Report

**DOI:** 10.3400/avd.cr.22-00015

**Published:** 2022-06-25

**Authors:** Kazuki Noda, Koki Yokawa, Naoki Dan, Hitoshi Matsuda

**Affiliations:** 1 Department of Cardiovascular Surgery, National Cerebral and Cardiovascular Center, Suita, Osaka, Japan; 2 Department of Gastroenterology, Suita Municipal Hospital, Suita, Osaka, Japan

**Keywords:** primary aortoduodenal fistula, endovascular procedure, endoscopic treatment

## Abstract

Primary aortoduodenal fistula (ADF) is a relatively rare and morbid diagnosis. A 91-year-old man who developed hematemesis and melena was transferred from a community hospital with the diagnosis of a ruptured abdominal aortic aneurysm (AAA). Computed tomography revealed an irregular-shaped AAA with cavities enhanced near the duodenum, with suspected ADF. The patient was initially treated with emergency endovascular aneurysm repair. Duodenoscopy showed defects of the mucosa. ADF was diagnosed, and fistulas were closed with endoscopic clipping. This case highlights the success of ADF endovascular repair.

## Introduction

Primary aortoduodenal fistula (ADF) is a relatively rare and morbid diagnosis. The efficacy of endovascular repair instead of surgical repair has recently been reported. However, the high risk of aortic infection due to the placing of engrafts remains a major concern. Herein, a successful case of endovascular and endoscopic treatment for primary ADF, controlling endograft infection, is reported.

## Case Report

**Figure 1:**
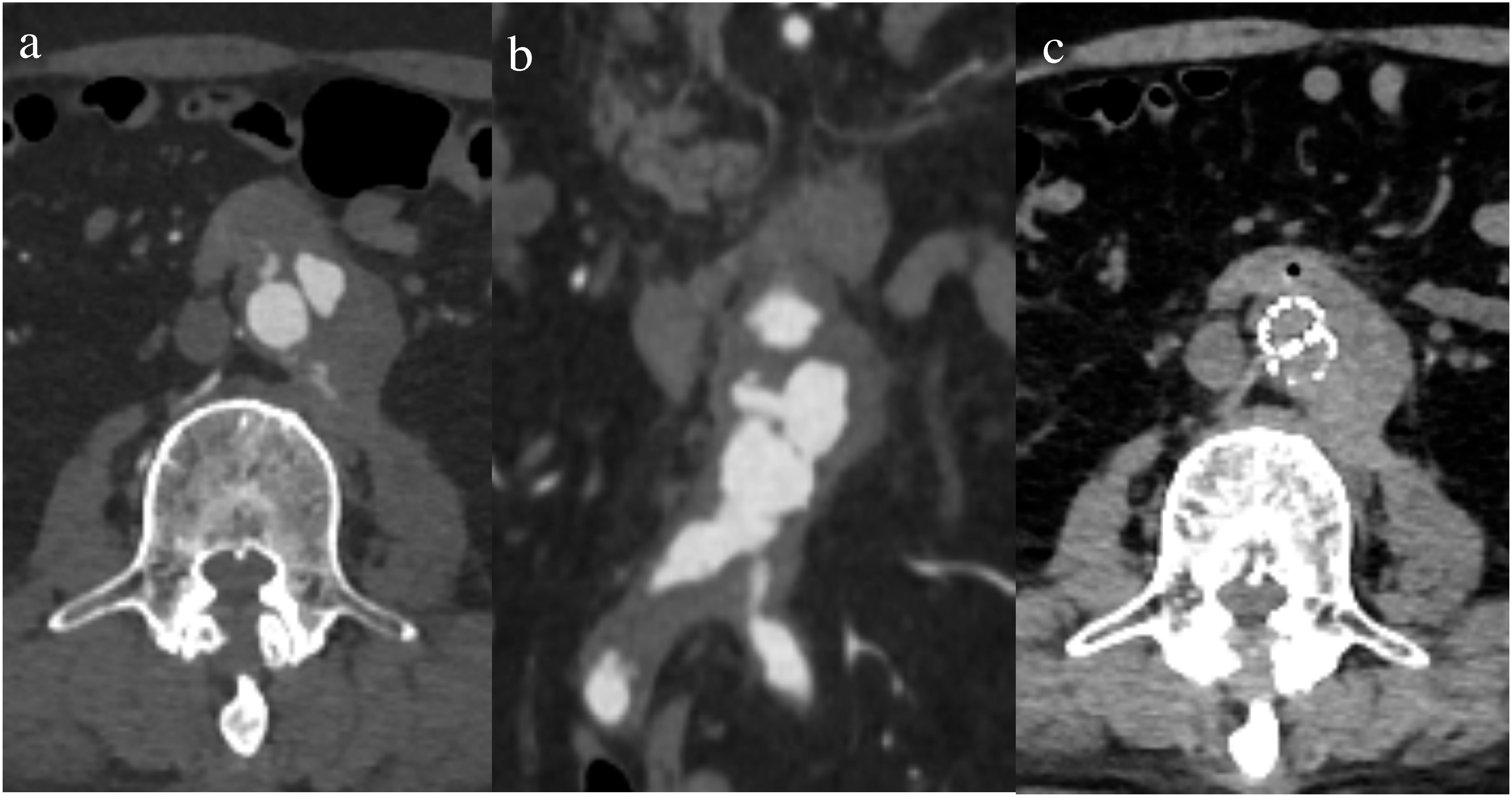
Fig. 1 Computed tomography angiography revealed that the horizontal (**a**) and coronal (**b**) portions of the duodenum were close to an abdominal aortic aneurysm without leakage of Gastrografin after endovascular aneurysm repair (**c**).

A 91-year-old man with severe frailty who had developed hematemesis and melena twice in 4 weeks was transferred from a community hospital with the diagnosis of a ruptured abdominal aortic aneurysm (AAA). Computed tomography (CT) revealed an irregular-shaped AAA with surrounding low-density area extended to the left psoas muscle at the dorsal side and cavities enhanced with dye inside the low-density area near the duodenum. No septum was detected between the aneurysm and duodenum, and ADF was suspected. However, no dye extravasation was found inside the duodenum (**Figs. 1a** and **1b**, Video 1). Laboratory data revealed no evidence of infection; the white blood cell count was 4,050/mm^3^ and C-reactive protein was 0.31 mg/dl. Two sets of blood culture were negative at the time of hospitalization. Considering the patient’s age and frailty, emergency endovascular aneurysm repair (EVAR) with the Endurant Endograft (Medtronic Inc., Santa Rosa, CA, USA) was indicated to treat the state of rupture.

**Figure 2:**
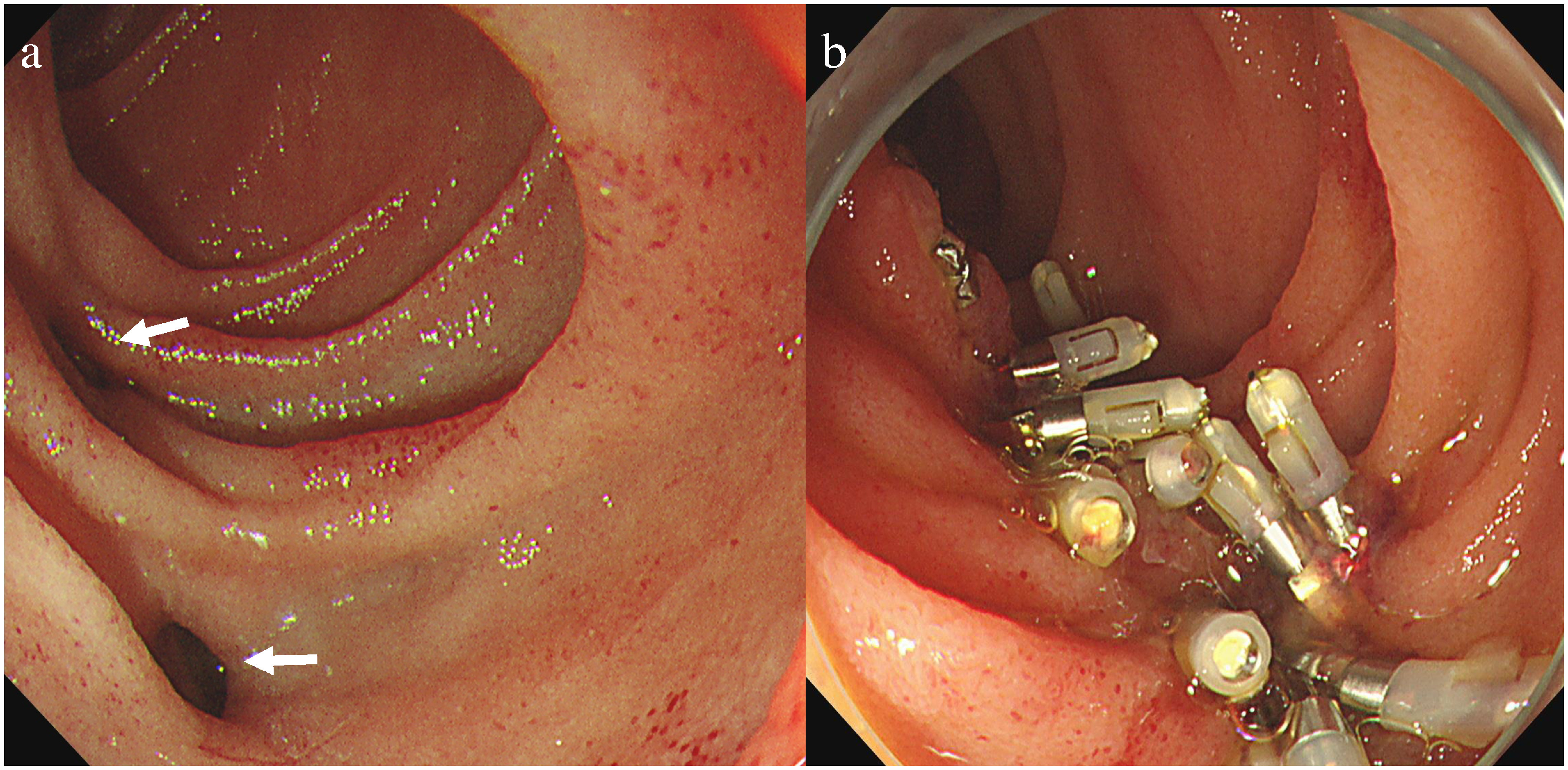
Fig. 2 Duodenoscopy demonstrated fistulae (**a**) in the duodenum (white arrows), and endoscopic clippings were indicated (**b**).

The patient remained afebrile without recurrent hematemesis or melena 2 weeks after EVAR, with broad-spectrum antibiotic therapy and fasting with total parenteral nutrition. CT with the oral intake of Gastrografin (Bayer, Osaka, Japan) revealed no leakage to the aneurysm sac (**Fig. 1c**), but the duodenoscopy demonstrated two areas of mucosal defect without exposure of the endograft and bleeding (**Fig. 2a**). These findings suggested small perforations, and endoscopic clippings could be performed successfully to close the fistulae (**Fig. 2b**). Antibiotic therapy was halted and oral liquid diet was started the next day. The patient was discharged home 42 days after EVAR and has been followed-up for 6 months at an outpatient clinic without the recurrence of ADF.

## Discussion

Primary ADF is a rare cause of gastrointestinal bleeding with an incidence rate of <0.2%, causing 1%–2% of the aortic aneurysm complications.^1,2)^ Open surgery is a primary ADF therapeutic approach but is associated with significant morbidity and mortality. EVAR is less invasive, provides rapid aneurysm exclusion, and achieves prompt bleeding control if the aneurysm is anatomically feasible for EVAR. However, its indication is limited to specific situations (e.g., hemodynamic instability) in patients with frailty or bridge treatment to open surgery due to the significant possibility of graft contamination.^3)^

Baril et al. reported a successful combination of endovascular repair and long-term antibiotic therapy for aortoenteric fistula.^4)^ The patient reported herein was a nonagenarian and revealed no marked infection signs; the EVAR indication could be justified along with antibiotic therapy and intestinal decompression by fasting.

Duodenoscopy was indicated for the safe restart of food intake and revealed limited mucosa defects. This finding led to the definite ADF diagnosis, but direct exposure of the endograft to the enteric cavity was not observed. Endoscopic clipping was indicated, which has been proven to control bleeding and close fistulas and small perforations.^5)^ Although Fan et al. reported the closure of a 1-cm duodenal wall perforation,^6)^ there is no definite limitation in the size of closable perforations. The fistula with active bleeding is also inappropriate for endoscopic clippings.

No definite indication about the period of ADF antibiotic therapy, especially in patients with artificial grafts, was noted and long-term antibiotic administration is usually unavoidable. In this case, antibiotic therapy was halted after endoscopic clippings because of no evidence of infection. The ADF cure in this report with endoscopic closure might also reduce the length of antibiotic therapy.

## Conclusion

A successful endovascular and endoscopic primary ADF treatment was reported herein.

## Supplementary Information

Supplementary movie is available at the online article sites on J-STAGE and PMC.

## References

[1] Beuran M, Negoi I, Negoi RI, et al. Primary aortoduodenal fistula: first you should suspect it. Braz J Cardiovasc Surg 2016; 31: 261-3. 27737411 10.5935/1678-9741.20160049 PMC5062716

[2] Bissacco D, Freni L, Attisani L, et al. Unusual clinical presentation of primary aortoduodenal fistula. Gastroenterol Rep (Oxf) 2015; 3: 170-4. 24982129 10.1093/gastro/gou040 PMC4423455

[3] Setacci C, de Donato G, Setacci F. Endografts for the treatment of aortic infection. Semin Vasc Surg 2011; 24: 242-9. 22230680 10.1053/j.semvascsurg.2011.10.009

[4] Baril DT, Carroccio A, Ellozy SH, et al. Evolving strategies for the treatment of aortoenteric fistulas. J Vasc Surg 2006; 44: 250-7. 16890849 10.1016/j.jvs.2006.04.031

[5] Grupka MJ, Benson J. Endoscopic clipping. J Dig Dis 2008; 9: 72-8. 18419639 10.1111/j.1751-2980.2008.00325.x

[6] Fan CS, Soon MS. Repair of a polypectomy-induced duodenal perforation with a combination of hemoclip and band ligation. Gastrointest Endosc 2007; 66: 203-5. 17521637 10.1016/j.gie.2006.11.025

